# Supplementary material for: Purkinje cell dopaminergic inputs to astrocytes regulate cerebellar-dependent behavior
Source: Nat Commun. 2023 Mar 23;14:1613. doi: 10.1038/s41467-023-37319-w (PMC10036610; doi:10.1038/s41467-023-37319-w)
Supplement: Supplementary file 1 — Supplementary Information [file 41467_2023_37319_MOESM1_ESM.pdf]

Supplementary Information

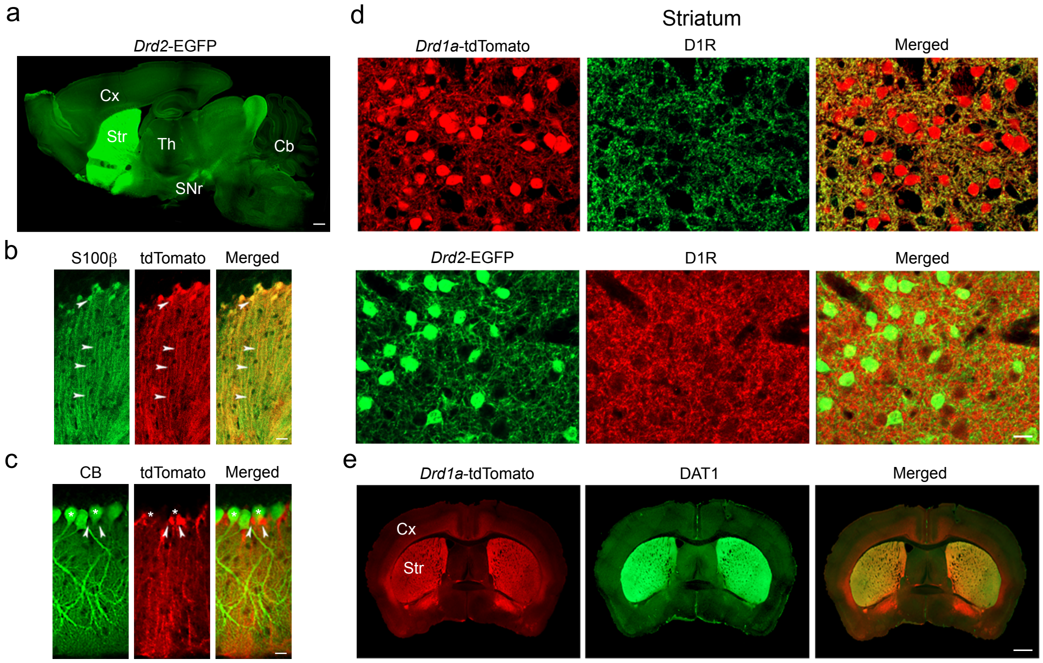

Supplementary Fig. 1 Characterization of D1R and D2R expression in the cerebellum and of D1R and DAT1 antibodies

a A sagittal brain section from a *Drd2*-EGFP mouse. Three biological replicates were performed. Cx, cerebral cortex; Str, striatum; Th, thalamus; SNr, substantia nigra pars reticulata; Cb, cerebellum. Scale bar, 500 μm. **b** Dual immunostaining of s100β and tdTomato. Arrowheads indicate colocalization. Five biological replicates were performed. Scale bar, 20 μm. **c** Dual immunostaining of CB and tdTomato. Arrowheads indicate BGs, and asterisks indicate PCs. Five biological replicates were performed. Scale bar, 20 μm. **d** In striatal sections, *Drd1a*-tdTomato is colocalized with D1R immunoreactivity (upper), whereas *Drd2*-EGFP is not (lower). Two biological replicates were performed. Scale bar, 20 μm. **e** DAT immunostaining in coronal brain sections shows that DAT1 is expressed in the striatum of *Drd1a*-tdTomato mice. Two biological replicates were performed. Scale bar, 400 μm.

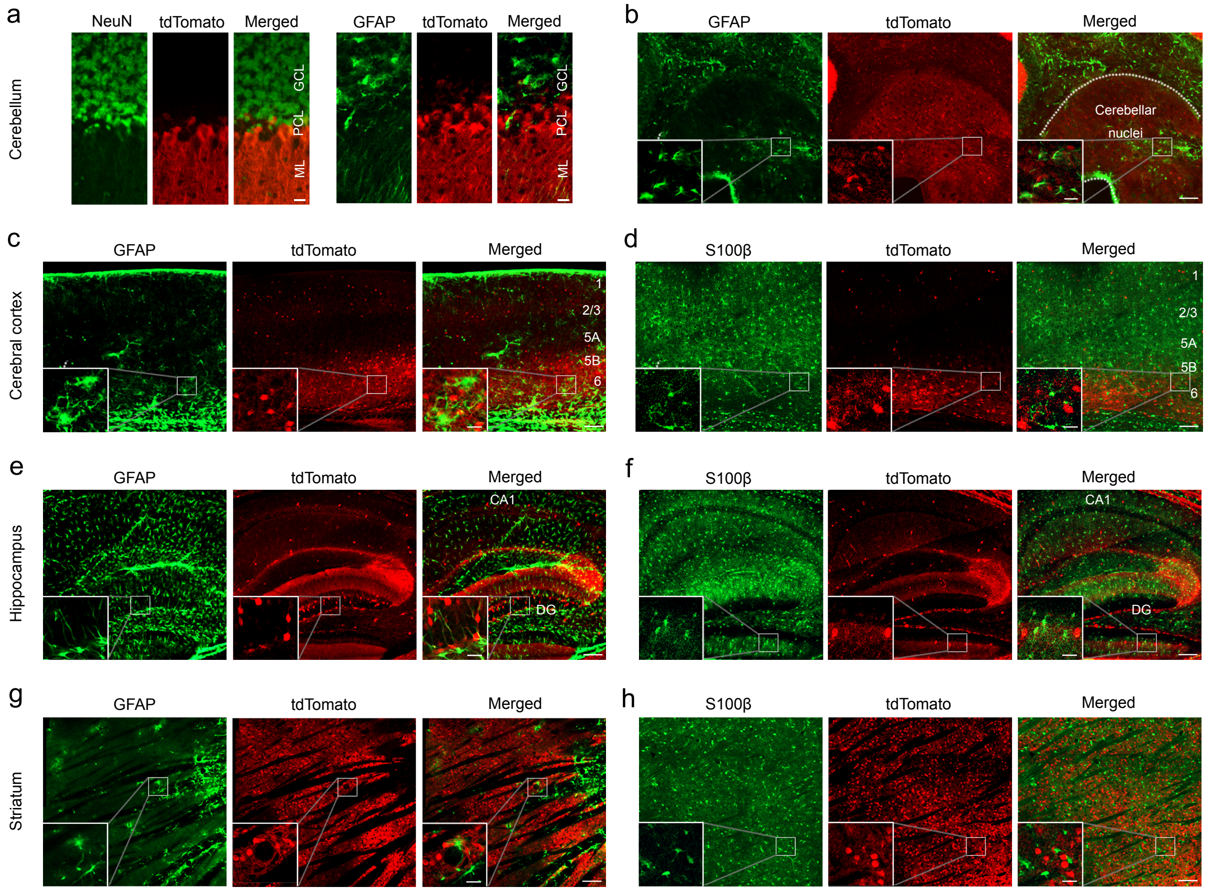

Supplementary Fig. 2 Characterization of D1R expression in several major brain regions

a Dual immunostaining of NeuN or GFAP and tdTomato in the cerebellum. No colocalization was observed. Five biological replicates were performed. GCL, granule cell layer; PCL, Purkinje cell layer; ML, molecular layer. Scale bar, 20 μm. **b** Dual immunostaining of GFAP and tdTomato in the cerebellar nuclei shows that tdTomato-expressing neurons are not colocalized with GFAP-expressing astrocytes. Five biological replicates were performed. Scale bar, 200 μm (inset, 40 μm). **c,d** Dual immunostaining of GFAP or s100β and tdTomato in the cerebral cortex shows that tdTomato-expressing neurons are not colocalized with GFAP- or s100β-expressing astrocytes. Three biological

replicates were performed for each. Scale bar, 200 µm (inset, 40 µm). **e,f** Dual immunostaining of GFAP or s100β and tdTomato in the hippocampus shows that tdTomato-expressing neurons are not colocalized with GFAP- or s100β-expressing astrocytes. CA1, cornu ammonis area 1; DG, dentate gyrus. Three biological replicates were performed for each. Scale bar, 200 µm (inset, 40 µm). **g,h** Dual immunostaining of GFAP or s100β and tdTomato in the striatum shows that tdTomato-expressing neurons are not colocalized with GFAP- or s100β-expressing astrocytes. Three biological replicates were performed for each. Scale bar, 200 µm (inset, 40 µm).

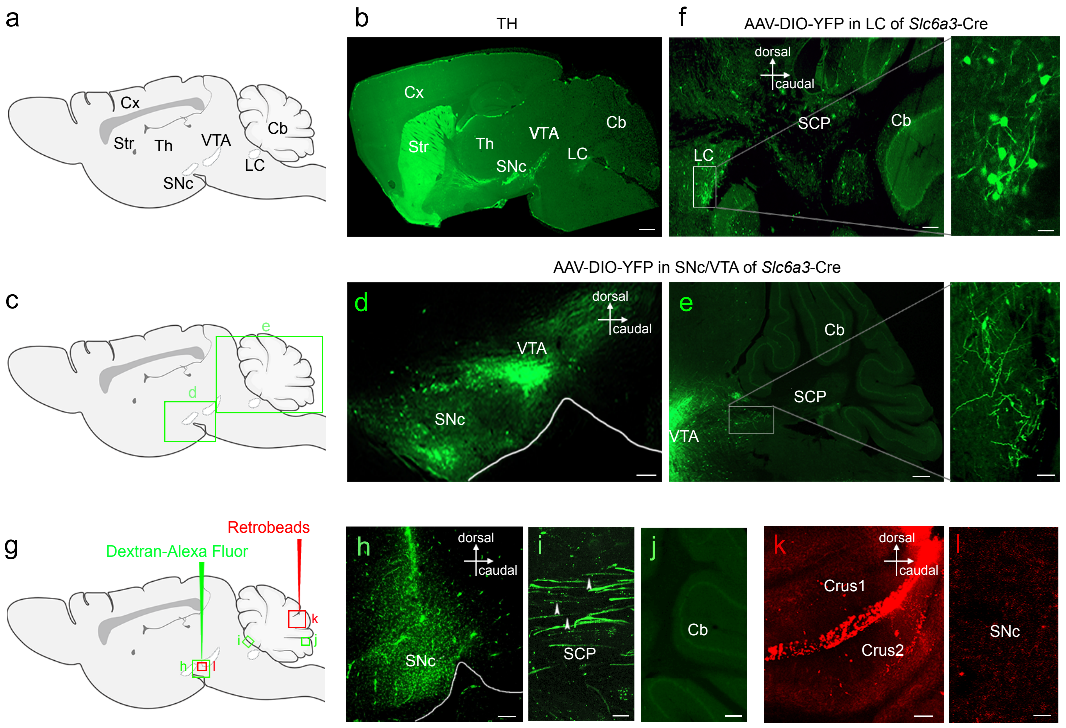

Supplementary Fig. 3 Possible sources of DA in the cerebellum

a,b TH immunostaining in a sagittal brain section. The schematic shows brain regions (a). Three biological replicates were performed. Cx, cerebral cortex; Str, striatum; Th, thalamus; SNc, substantia nigra pars compacta; VTA, ventral tegmental area; LC, locus coeruleus; Cb, cerebellum. Scale bar, 500 μm. **c-e** YFP immunostaining in the VTA and SNc. The schematic shows the imaged regions (c). AAV-DIO-YFP was injected into the SNc and VTA of *Slc6a3*-Cre mice for expression of YFP (d). Imaging in the near superior cerebellar peduncle (SCP; e, white box) shows that axonal fibers from the VTA or SNc enter the deep cerebellum nuclei. Three biological replicates were performed. Scale bar, 400 μm (d and e; enlarged image, 50 μm). **f** YFP immunostaining in the LC. AAV-DIO-YFP was injected into the LC of *Slc6a3*-Cre mice for expression of YFP. Three biological replicates were performed. Scale bar, 200 μm (enlarged image, 50 μm). **g-l** Antegrade and retrograde tracing between the midbrain and cerebellum. The schematic shows

injections of Dextran-Alexa Fluor 488 (500 nl) in the SNc, and retrobeads (200 nl) in the cerebellar crus 1 and 2 (g). Green boxes indicate imaging in the SNc (h), SCP (i), and cerebellar cortex (j) following the injection of Dextran-Alexa Fluor 488; red boxes indicate imaging in the cerebellar cortex (k) and SNc (l) following the injection of retrobeads. No apparent labeling was seen in the cerebellar cortex following injections of Dextran-Alexa Fluor 488 or in the SNc/VTA following injections of retrobeads. Three biological replicates were performed. Scale bar, 200 μm (h, j, k) and 50 μm (i, l). The cartoons in panels a, c, and g were created with BioRender.com.

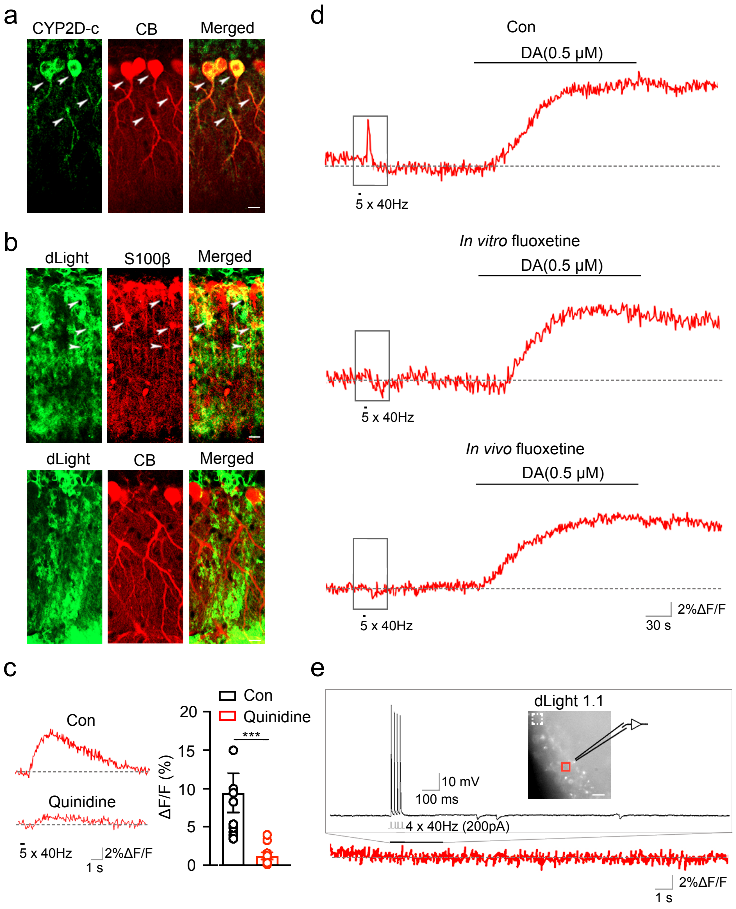

Supplementary Fig. 4 CAG-dLight1.1 expression and dLight1.1 signals in the cerebellum

a Dual immunostaining of CYP2D and CB. Arrowheads indicate colocalization of CYP2D6 and CB. An antibody against the C terminal region of CYP2D6 was used. Scale bar, 20 μm. Two biological replicates were performed**. b** Dual immunostaining of dLight1.1-GFP and S100β or CB. CAG-dLight1.1-GFP was expressed in the cerebellum. Arrowheads indicate colocalization of dLight1.1 and S100β (upper), but not of dLight1.1 and CB (lower). Four biological replicates were performed. Scale bar, 20 μm. **c** dLight1.1 signals evoked in slices perfused with normal aCSF (n = 10 slices/3 mice) and in slices pretreated with quinidine (50 μM) for 2 h (n = 10 slices/3 mice). Data are presented as mean ± SEM.

Mann-Whitney two-sided test ($p < 0.0001$, Con *vs.* Quinidine). **d** dLight1.1 signals evoked by electrical stimulus followed by DA application in control slices, in slices pretreated with fluoxetine, or in slices from animals administered with fluoxetine (n = 2 slices/2 mice, each group). Boxes indicate dLight1.1 signals in control slices but no signals in two groups treated with fluoxetine. **e** Absence of dLight1.1 signals in the cerebellum (n = 6 slices/3 mice) following the firing of an adjacent PC evoked by current injections at resting membrane potentials (200 pA, 4 pulses at 25 Hz). Inset shows four evoked action potentials in a PC (left) and the locations of electrode pipette placement for current injections and recording of dLight1.1 signals. The solid red box shows ROIs where dLight1.1 signals were measured; the dashed blue box indicates background signals that were subtracted.

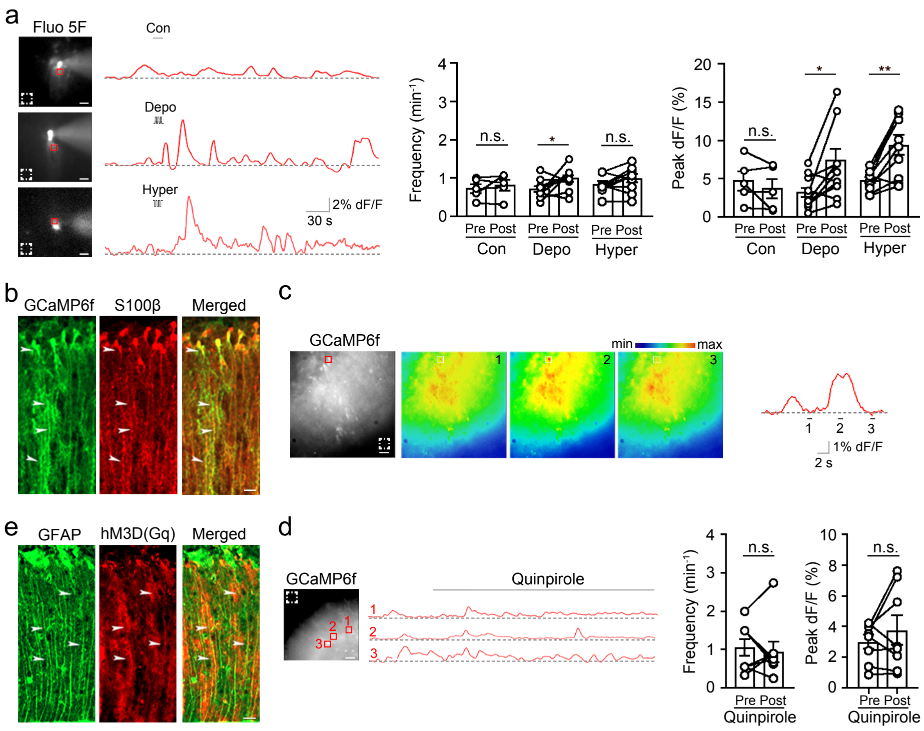

Supplementary Fig. 5 Ca^{2+} signals in Fluo 5F-filled and GCaMP6f-expressing BGs

a Ca^{2+} signals recorded in BGs filled with Fluo 5F in response to membrane depolarization to −20 mV or hyperpolarization to −120 mV three times at 0.1 Hz with 1-s duration. Both membrane depolarization and hyperpolarization enhanced the frequency and/or peak amplitude of Ca^{2+} signals (n = 5 slices/3 mice, Con; n = 11 slices/4 mice, Depo; n = 10 slices/4 mice, Hyper). The solid red box shows ROIs where Fluo 5F signals were measured; the dashed box indicates background signals that were subtracted. Scale bar, 20 μm. Data are presented as mean ± SEM. Paired two-sided student's *t* test ($p = 0.4569$, Frequency Con; $p = 0.0303$, Frequency Depo; $p = 0.0680$, Frequency Hyper; $p = 0.2426$, Peak dF/F Con; $p = 0.0107$, Peak dF/F Depo; $p = 0.0015$, Peak dF/F Hyper). **b** Colocalization of GCaMP6f-GFP and S100β immunostaining (arrowheads). Four biological replicates were performed. Scale bar, 20 μm. **c** Heat map showing spontaneous Ca^{2+} signals in GCaMP6f-expressing BGs. The solid red box shows ROIs where

GCaMP6f signals were measured; the dashed box indicates background signals that were subtracted. Three biological replicates were performed. Minimal and maximum values indicate the relative amplitude of Ca^{2+} signals recorded from the slice. Scale bar, 50 μm. **d** Ca^{2+} signals recorded in GCaMP6f-expressing BGs treated with quinpirole (n = 8 slices/4 mice). The solid red box shows ROIs where GCaMP6f signals were measured; the dashed box indicates background signals that were subtracted. Data are presented as mean ± SEM. Paired two-sided student's *t* test (p = 0.6377, Frequency; p = 0.3249, Peak dF/F Con). Scale bar, 50 μm. **e** Colocalization of GFAP and GFAP-hM3D(Gq)-mCherry immunostaining (arrowheads). Four biological replicates were performed. Scale bar, 20 μm. n.s., not significant.

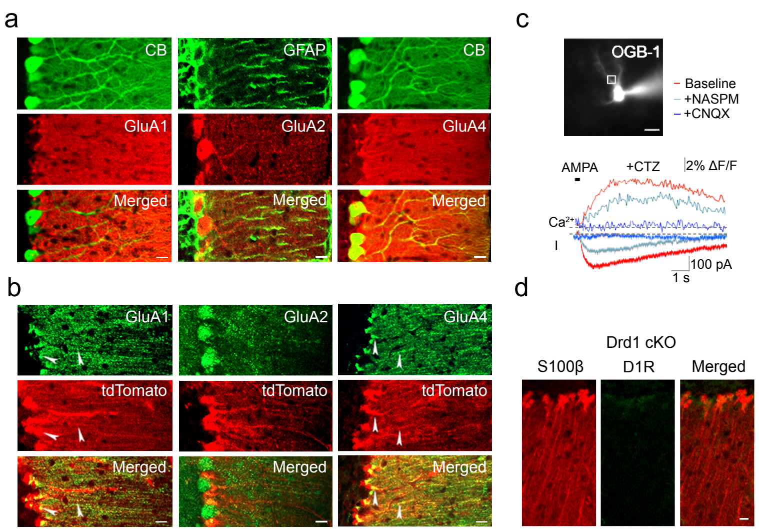

Supplementary Fig. 6 AMPAR subunits in the control or *Drd1* cKO mice

a Dual immunostaining of CB and GluA1, GFAP and GluA2, and CB and GluA4. Four biological replicates were performed. Scale bar, 20 μm. **b** Dual immunostaining of GluA1, GluA2, or GluA4, and tdTomato. Arrowheads indicate colocalization. Four biological replicates were performed. Scale bar, 20 μm. **c** Simultaneous Ca^{2+} imaging and whole-cell recordings in BGs filled with OGB-1 during treatments with NAPSM and CNQX (n = 2 slices/1 mouse). The solid red box shows ROIs where OGB-1 signals were measured; the dashed box indicates background signals that were subtracted. Two biological replicates were performed. Scale bar, 50 μm. **d** Dual immunostaining of S100β and D1R in cerebellar sections of *Drd1* cKO mice. Four biological replicates were performed. Scale bar, 20 μm.

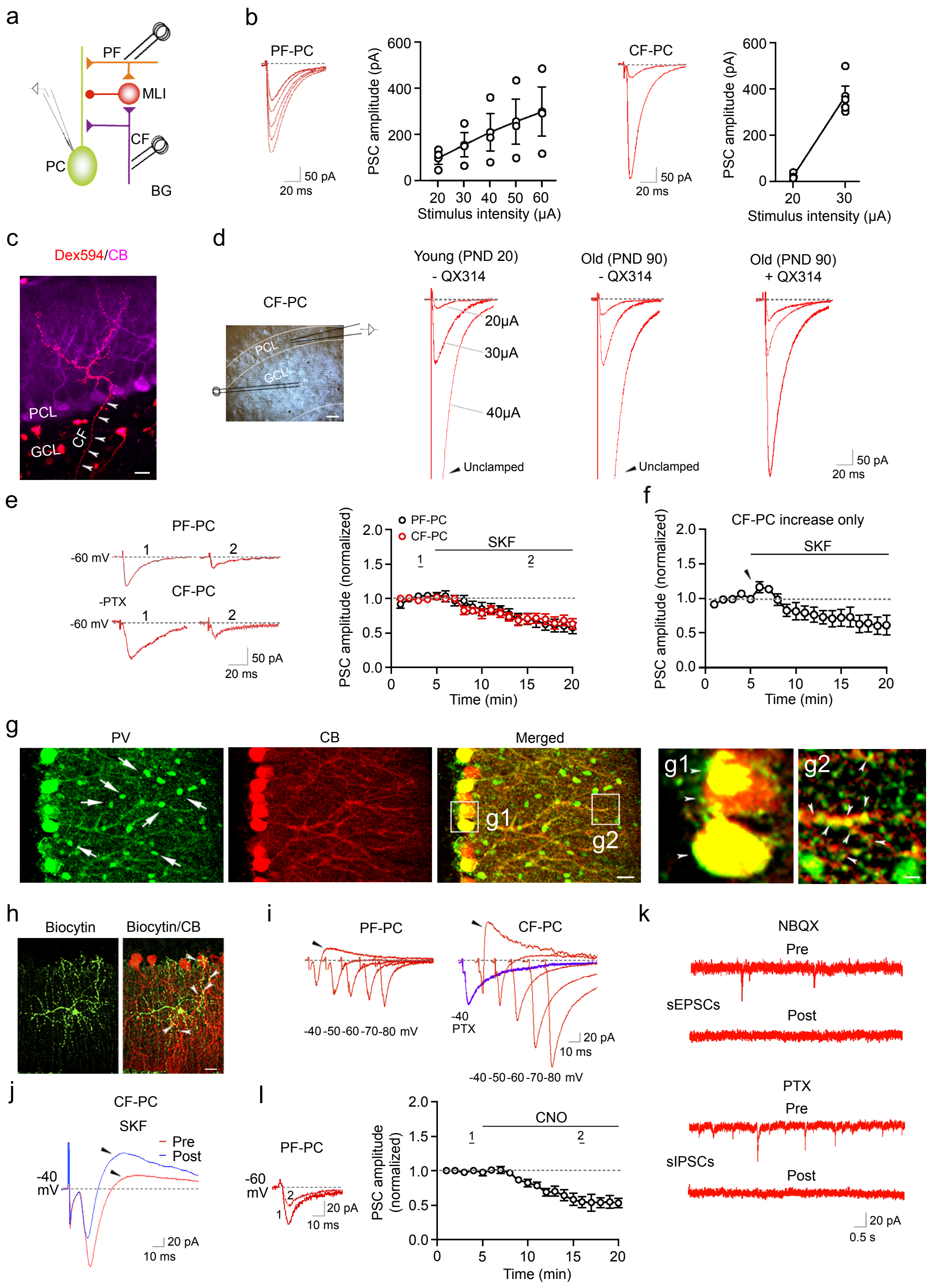

Supplementary Fig. 7 D1R activation reduces glutamatergic signaling but promotes GABAergic signaling onto PCs

a Diagram of whole-cell recordings in PCs and stimulation at PFs or CFs. BG, Bergmann glial cell; PF, parallel fiber; CL, climbing fiber; PC, Purkinje cell; MLI, molecular layer interneuron. **b** Input and output relationship between the stimulus intensity and the PSC amplitude at PF-PC (n = 3 slices/2 mice) and CF-PC (n = 4 slices/2 mice) synapses. Stimuli at PFs evoked graded PSCs in PCs, while stimuli at CFs evoked nearly all-or-none PSCs. Data are presented as mean ± SEM. **c** CF filled with Dextran-Alexa Fluor 594, followed by CB immunostaining. Arrowheads indicate that a CF crosses the GCL and wraps the PC. GCL, granule cell layer; PCL, Purkinje cell layer. Scale Bar, 20 μm. **d** An image showing a whole-cell recording of a PC during CF stimulation. Three biological replicates were performed. Scale Bar, 20 μm. **d** An image showing a whole-cell recording of a PC during CF stimulation. Scale Bar, 50 μm. With the increase of stimulus intensity (20, 30, and 40 μA), evoked PSCs were rapidly unclamped in PCs from young (~ PND 20; n = 3 slices/2 mice) and old (~ PND 90; n = 4 slices/3 mice) mice in the absence of intracellular QX314. Large PSCs were reliably recorded in PCs in the presence of QX314. **e** PSCs recorded at PF-PC (n = 9 slices/4 mice) and CF-PC (n = 10 slices/4 mice) synapses in slices before and during SKF83822 application. Data are presented as mean ± SEM. Unpaired student's *t* test ($p < 0.0001$, PF-PC; $p < 0.0001$, CF-PC; baseline *vs.* 15 min after SKF83822 application). **f** A few recordings (n = 5 slices/3 mice) showed a transient increase in the amplitude of PSCs evoked at CF-PC synapses during SKF83822 treatment (arrowheads). Data are presented as mean ± SEM. **g** Dual immunostaining of PV and CB. Arrows indicate PV-positive interneurons. Boxes are enlarged on the right, indicating that presynaptic terminals of MLIs synapse on the soma (g1) and dendrites (g2) of CB-labeled PCs. Five biological replicates were performed. Scale bar, 20 μm (lower

magnification) and 2 μm (higher magnification). **h** A MLI filled with biocytin, followed by CB immunostaining. Arrowheads indicate MLI axons innervate PCs. Three biological replicates were performed. Scale Bar, 40 μm. **i** PSCs recorded in PCs (at +80 to −40 mV) evoked by stimulation of PFs or CFs. Picrotoxin was added to demonstrate the outward inhibitory component of CF-PC PSCs at a holding potential of +40 mV. **j** Example traces show that SKF83822 enhanced outward IPSCs in a PC held at -40 mV (arrowheads) (n = 2 slices/1 mouse). **k** Example traces show sEPSCs (upper) and sIPSCs (lower), which were blocked by NBQX and picrotoxin, respectively. **l** PSCs recorded at PF-PC synapses in hM3D(Gq)-expressing slices (n = 9 slices/5 mice). Slices were treated with CNO. Data are presented as mean ± SEM. Mann-Whitney two-sided test (p = 0.0002, baseline *vs.* 15 min after CNO application). Example traces before (1) and during the treatment (2) are shown.

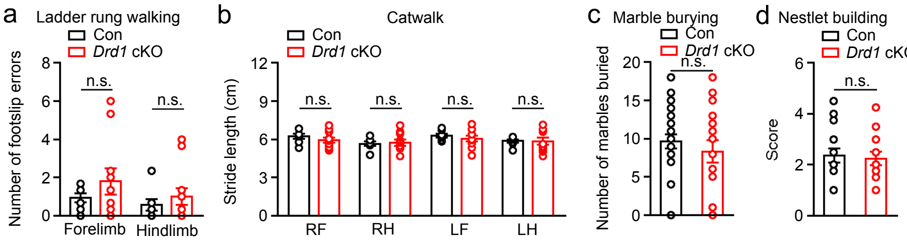

Supplementary Fig. 8 Behavioral analyses of *Drd1* cKO mice

a Foot-slip errors of forelimbs and hindlimbs on horizontal ladder rungs in control (n = 7) and *Drd1* cKO (n = 10, forelimb; n = 11, hindlimb) mice. Data are presented as mean ± SEM. Mann-Whitney two-sided test (p = 0.7865, Forelimb; p = 0.8767 Hindlimb; Con *vs. Drd1* cKO). **b** Stride length of four paws measured in control (n = 7) and *Drd1* cKO (n = 10) mice using CatWalk gait analysis system. RF, right forepaw; RH, right hindpaw; LF, left forepaw; LH, left hindpaw. Data are presented as mean ± SEM. Unpaired two-sided student's t test (p = 0.3514, RF; p = 0.8104 RH; p = 0.4383 LF; p = 0.9536 LH; Con *vs. Drd1* cKO). **c** The number of marbles buried by control (n = 22) and *Drd1* cKO (n = 16) mice. Data are presented as mean ± SEM. Unpaired two-sided student's t test (p = 0.4328, Con *vs. Drd1* cKO). **d** Nest quality score evaluated in control (n = 15) and *Drd1* cKO (n = 14) mice. Data are presented as mean ± SEM. Unpaired two-sided student's t test (p = 0.7685, Con *vs. Drd1* cKO). n.s., not significant.

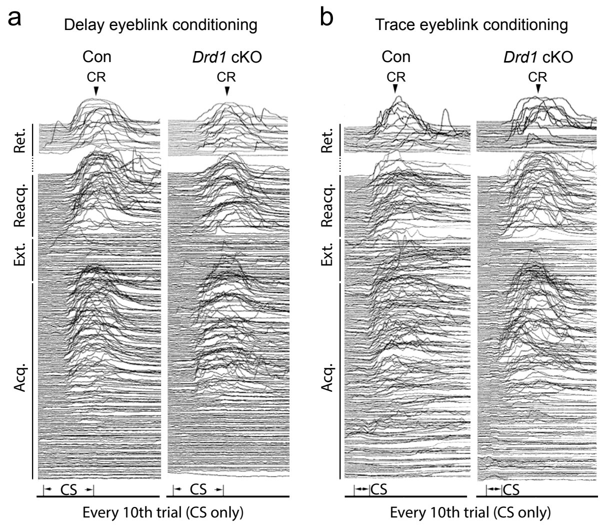

Supplementary Fig. 9 CR amplitude in eyeblink conditioning

a,b CR amplitude in delay (a) and trace (b) eyeblink conditioning in control and *Drd1* cKO mice. CR amplitude was measured by fraction eyelid closure from every 10th trial in acquisition, extinction, reacquisition, and retention phases.

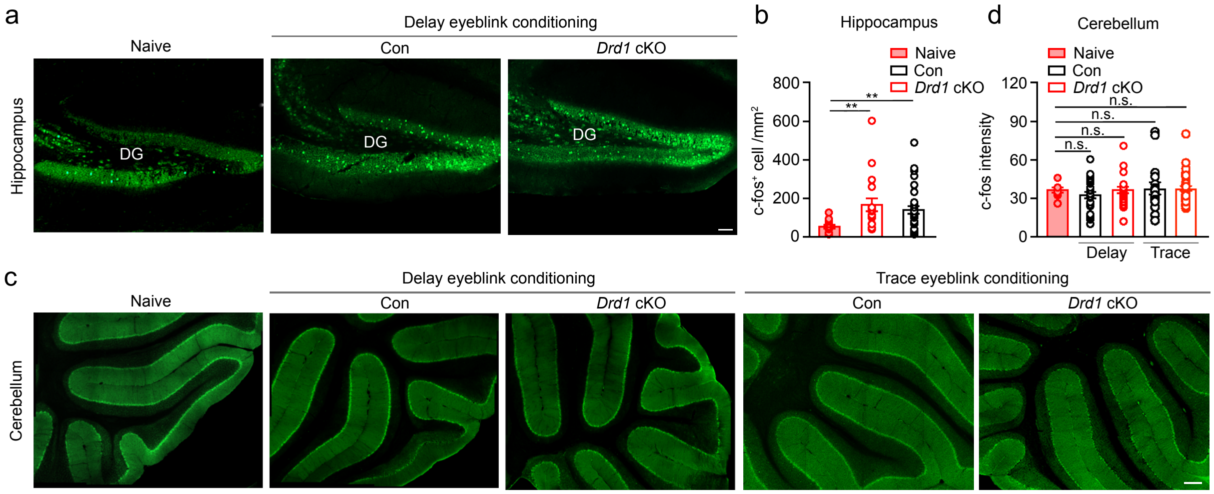

Supplementary Fig. 10 Cell activity after eyeblink conditioning measured by c-fos in the hippocampus and cerebellum

a Immunostaining of c-fos in the hippocampus of native mice, and control and *Drd1* cKO mice that received delay eyeblink conditioning. One hour after the last session of eyeblink conditioning, mice were perfused with 4% paraformaldehyde and processed for immunostaining of c-fos. Naive mice were kept in a home cage under the same light condition as control and *Drd1* cKO mice until fixative perfusion. As the DG has the most c-fos$^+$ cells following conditioning, only this region was evaluated for cell number. **b** Average number of c-fos$^+$ cells in the dentate gyrus (DG) of the hippocampus (n = 15 sections/4 mice, Naïve; n = 33 sections/9 mice, Con; n = 18 sections/4 mice, *Drd1* cKO). Scale bar, 100 μm. Data are presented as mean ± SEM. Multiple unpaired two-sided student's *t* test (p = 0.0049, Naïve *vs.* Con; p = 0.0087, Naïve *vs. Drd1* cKO). **c** Immunostaining of c-fos in the cerebellum of native mice, and control and *Drd1* cKO mice that received delay or trace eyeblink conditioning. **d** Average intensity of c-fos$^+$ cells in the cerebellum following delay (n = 12 sections/4 mice, Naïve; n = 26 sections/9 mice; Con n = 24 sections/4 mice; *Drd1* cKO) and trace (n = 20 sections/5 mice, Con; n = 43

sections/9 mice, *Drd1* cKO) eyeblink conditioning. Scale bar: 400 μm. Data are presented as mean ± SEM. Multiple unpaired two-sided student's *t* test (p = 0.3422, Delay Naïve *vs.* Con; p = 0.9900, Delay Naïve *vs. Drd1* cKO; p = 0.8588, Trace Naïve *vs.* Con; p = 0.7345, Trace Naïve *vs. Drd1* cKO). n.s., not significant.

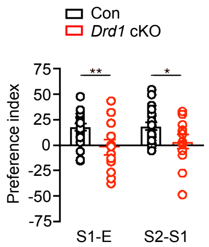

Supplementary Fig. 11 Social interaction evaluated by preference index

Preference index shows the relative time that mice spent with the empty cup and other mice (n = 22, Con; n = 12, *Drd1* cKO). It is defined as the numerical difference between S1 and E, or S2 and S1 relative to the sum of S1 and E, or S1 and S2, multiplied by 100, respectively. Data are presented as mean ± SEM. Unpaired two-sided student's *t* test (p = 0.0099, S1-E; p = 0.0491, S2-S1; Con *vs. Drd1* cKO).
