# Supplementary material for: Purkinje cell dopaminergic inputs to astrocytes regulate cerebellar-dependent behavior
Source: Nat Commun. 2023 Mar 23;14:1613. doi: 10.1038/s41467-023-37319-w (PMC10036610; doi:10.1038/s41467-023-37319-w)
Supplement: Supplementary file 3 — Description of Additional Supplementary Files [file 41467_2023_37319_MOESM3_ESM.pdf]

Description of Additional Supplementary Files

Supplementary Movie 1: Ca^{2+} dynamics in GCaMP6f-expressing BGs treated with SKF83822.
Ca^{2+} signals recorded for 10 min in BGs. SKF83822 was bath-applied following the baseline recording for 2 min.

Supplementary Movie 2: **Ca^{2+} dynamics in GCaMP6f-expressing BGs treated with SKF83822 in the presence of 2-APB.**
Ca^{2+} signals recorded for 10 min in BGs incubated with 2-APB. SKF83822 was bath-applied following the baseline recording for 2 min.
