# Supplementary material for: Purkinje cell dopaminergic inputs to astrocytes regulate cerebellar-dependent behavior
Source: Nat Commun. 2023 Mar 23;14:1613. doi: 10.1038/s41467-023-37319-w (PMC10036610; doi:10.1038/s41467-023-37319-w)
Supplement: Supplementary file 6 — Reporting Summary [file 41467_2023_37319_MOESM6_ESM.pdf]

nature portfolio

Corresponding author(s): Wei Li

Last updated by author(s): Jan 31, 2023

Reporting Summary

Nature Portfolio wishes to improve the reproducibility of the work that we publish. This form provides structure for consistency and transparency in reporting. For further information on Nature Portfolio policies, see our Editorial Policies and the Editorial Policy Checklist.

Statistics

For all statistical analyses, confirm that the following items are present in the figure legend, table legend, main text, or Methods section.

n/a	Confirmed	
☐	☒	The exact sample size (*n*) for each experimental group/condition, given as a discrete number and unit of measurement
☐	☒	A statement on whether measurements were taken from distinct samples or whether the same sample was measured repeatedly
☐	☒	The statistical test(s) used AND whether they are one- or two-sided<br>*Only common tests should be described solely by name; describe more complex techniques in the Methods section.*
☒	☐	A description of all covariates tested
☐	☒	A description of any assumptions or corrections, such as tests of normality and adjustment for multiple comparisons
☐	☒	A full description of the statistical parameters including central tendency (e.g. means) or other basic estimates (e.g. regression coefficient) AND variation (e.g. standard deviation) or associated estimates of uncertainty (e.g. confidence intervals)
☐	☒	For null hypothesis testing, the test statistic (e.g. F, t, r) with confidence intervals, effect sizes, degrees of freedom and P value noted<br>*Give P values as exact values whenever suitable.*
☒	☐	For Bayesian analysis, information on the choice of priors and Markov chain Monte Carlo settings
☒	☐	For hierarchical and complex designs, identification of the appropriate level for tests and full reporting of outcomes
☒	☐	Estimates of effect sizes (e.g. Cohen's *d*, Pearson's *r*), indicating how they were calculated

Our web collection on statistics for biologists contains articles on many of the points above.

Software and code

Policy information about availability of computer code

Data collection	TI Workbench (electrophysiology); LSM-800 Airyscan confocal microscope (immunofluorescence); EthoVision XT 16 (motor and social behavior); MATLAB 2018a, 2021a (eyeblink conditioning; https://zenodo.org/record/7569717#.Y9lIy-zMLzc)
Data analysis	AQuA software (Ca2+ signals); GarphPad Prism 8, 9 (Statistics)

For manuscripts utilizing custom algorithms or software that are central to the research but not yet described in published literature, software must be made available to editors and reviewers. We strongly encourage code deposition in a community repository (e.g. GitHub). See the Nature Portfolio guidelines for submitting code & software for further information.

Data

Policy information about availability of data

All manuscripts must include a data availability statement. This statement should provide the following information, where applicable:

- Accession codes, unique identifiers, or web links for publicly available datasets
- A description of any restrictions on data availability
- For clinical datasets or third party data, please ensure that the statement adheres to our policy

Source data are provided with this paper. All data are available upon request to the corresponding author.

Human research participants

Policy information about studies involving human research participants and Sex and Gender in Research.

Reporting on sex and gender	n/a
Population characteristics	n/a
Recruitment	n/a
Ethics oversight	n/a

Note that full information on the approval of the study protocol must also be provided in the manuscript.

Field-specific reporting

Please select the one below that is the best fit for your research. If you are not sure, read the appropriate sections before making your selection.

☒ Life sciences ☐ Behavioural & social sciences ☐ Ecological, evolutionary & environmental sciences

For a reference copy of the document with all sections, see nature.com/documents/nr-reporting-summary-flat.pdf

Life sciences study design

All studies must disclose on these points even when the disclosure is negative.

Sample size	We did not perform power analysis to predetermine sample size, but our sample size was based on previous experience and published results from slice or behavioral experiments (Li et al. Proc Natl Acad Sci USA: 13(11):E1575-84(2016); Li et al. Proc Natl Acad Sci USA109:17087-92 (2012)). We followed the recommendations of the IACUC of the University of Alabama at Birmingham by minimizing the use of animals.
Data exclusions	No data points were excluded from the analyses.
Replication	The number of independently replicated experiments has been described in the figure legends. All attempts to replicate the results were successful.
Randomization	Ex vivo slices or brain tissues were randomly assigned in electrophysiological recordings, dopamine sensor or Ca2+ imaging, and Western blotting. All control or conditional knockout mice were randomly assigned to different groups in all behavioral experiments.
Blinding	All behavioral experiments were performed blinded to the genotypes and treatment groups. For electrophysiology and imaging, as each recording severed as its own control, no blinding was necessary.

Reporting for specific materials, systems and methods

We require information from authors about some types of materials, experimental systems and methods used in many studies. Here, indicate whether each material, system or method listed is relevant to your study. If you are not sure if a list item applies to your research, read the appropriate section before selecting a response.

Materials & experimental systems

n/a	Involved in the study	
☐	☒	Antibodies
☒	☐	Eukaryotic cell lines
☒	☐	Palaeontology and archaeology
☐	☒	Animals and other organisms
☒	☐	Clinical data
☒	☐	Dual use research of concern

Methods

n/a	Involved in the study	
☒	☐	ChIP-seq
☒	☐	Flow cytometry
☒	☐	MRI-based neuroimaging

Antibodies

Antibodies used	Mouse monoclonal anti-GFAP (Sigma-Aldrich Cat#: G3893);<br>Rabbit living colors polyclonal anti-DsRed (TaKaRa Cat#: 632496);<br>Mouse monoclonal anti-RFP (Rockland Immunochemicals Cat#: 201-301-379);

Mouse anti-D1R, clone SG2-D1a (Sigma-Aldrich Cat#: MAB5290);
Rabbit monoclonal anti-D1R (Abcam Cat#: ab81296);
Mouse monoclonal anti-DAT (Thermo Fisher Scientific Cat#: MA5-24796);
Rabbit polyclonal anti-GFP (Abcam Cat#: ab290);
Chicken polyclonal anti-GFP (Abcam Cat#: ab13970);
Mouse monoclonal anti-calbindin D28K (Santa Cruz Biotechnology Cat#: sc-365360);
Rabbit polyclonal anti-CYP2D6 (Sigma-Aldrich Cat#: AV41675);
Rabbit polyclonal anti-CYP2D6 (Thermo Fisher Scientific Cat#: PAS-79129);
Mouse monoclonal anti-TH (Sigma-Aldrich Cat#: MAB318);
Rabbit polyclonal anti-mCherry (Abcam Cat#: ab167453);
Rabbit polyclonal anti-S100b (Sigma-Aldrich Cat#: HPA015768);
Rabbit polyclonal anti-GluA1 (Sigma-Aldrich Cat#: AB1504);
Rabbit polyclonal anti-GluA2 (Thermo Fisher Scientific Cat#: PA5-19496);
Mouse monoclonal anti-GluA2 (Thermo Fisher Scientific Cat#: MA5-17084);
Rabbit polyclonal anti-GluA4 (Sigma-Aldrich Cat#: AB1508);
Rabbit polyclonal anti-p831 GluA1 (Sigma-Aldrich Cat#: AB5847);
Rabbit polyclonal anti-p845 GluA1 (Thermo Fisher Scientific Cat#: OPA1-04118);
Guinea pig polyclonal anti-c-fos (Synaptic Systems Cat#: 226004);
Guinea pig polyclonal anti-PV (Synaptic Systems Cat#: 195004);
Rabbit monoclonal anti-mTOR (Cell Signaling Technology Cat#: 2983);
Rabbit monoclonal anti-p2448 mTOR (Cell Signaling Technology Cat#: 5536);
Mouse monoclonal anti-b-actin (Thermo Fisher Scientific Cat#: MA5-15739);
Streptavidin, Alexa Fluor 488 (Thermo Fisher Scientific Cat#: S32354);
Alexa Fluor 488 goat anti-mouse (Jackson ImmunoResearch Laboratories Cat#: 115-545-003);
Alexa Fluor 488 goat anti-rabbit (Jackson ImmunoResearch Laboratories Cat#: 111-545-003);
Alexa Fluor 488 goat anti-chicken (Jackson ImmunoResearch Laboratories Cat#: 103-545-155);
Alexa Fluor 488 goat anti-guinea pig (Jackson ImmunoResearch Laboratories Cat#: 106-545-003);
Alexa Fluor 594 goat anti-mouse (Jackson ImmunoResearch Laboratories Cat#: 115-585-003);
Alexa Fluor 594 goat anti-rabbit (Jackson ImmunoResearch Laboratories Cat#: 111-585-003);
Peroxidase-AffiniPure goat anti-rabbit (Jackson ImmunoResearch Laboratories Cat#: 111-035-003);
IRDye 800CW goat anti-mouse (LI-COR Biosciences Cat#: 926-32210).

Validation

Antibodies used in this study are commercially available and have been validated by us, the vendors or previous publications. Below is the relevant information for the primary antibodies.

Anti-GFAP: this antibody reacts specifically with GFAP in Bergmann glia cells (https://www.sigmaaldrich.com/US/en/product/sigma/g3893).
Anti-DsRed: this antibody detects tdTomato and mCherry and can be used for immunohistochemistry (De Luca et al. Nat Commun 13: 4163, (2022)).
Anti-RFP: applications include IF and ICH, and it reacts with mCherry and RFP (https://www.rockland.com/categories/primary-antibodies/rfp-antibody-200-301-379/).
Anti-D1R, clone SG2-D1a: it reacts specifically with mouse D1 receptors. It has been validated in our study in the striatum, where D1 receptors are known to be highly expressed (also see Liu et al. Mol Neurobiol 57: 4060, (2020)).
Anti-D1R: this antibody has been tested in mouse brain tissue shown on the manufacturer's website (https://www.abcam.com/dopamine-receptor-d1-antibody-ep1560y-ab81296.html#lb).
Anti-DAT: it reacts with DAT in mouse, rat, and human. It has been validated in our study in the striatum and in the mouse brain tissue shown on the manufacturer's website (https://www.thermofisher.com/antibody/product/Dopamine-Transporter-Antibody-clone-mAb16-Monoclonal/MA5-24796).
Rabbit anti-GFP: this antibody has been widely used in mice to detect recombinant GFP (see https://www.abcam.com/gfp-antibody-ab290.html).
Chicken anti-GFP: this antibody has been widely referenced; it reacts with recombinant GFP expressed in mouse tissue.
Anti-calbindin D28K: this antibody detects calbindin D28K, which has been tested in mouse tissue shown on the manufacturer's website (https://www.scbt.com/p/calbindin-d28k-antibody-d-4).
N-terminal anti-CyP2D6: it reacts with the mouse CyP2D6. This has been used for Western blotting and IHC (https://www.sigmaaldrich.com/US/en/product/sigma/av41675).
C-terminal anti-CyP2D6: this antibody has been successfully used to detect mouse CyP2D6, as shown on the manufacturer's website (https://www.thermofisher.com/antibody/product/CYP2D6-Antibody-Polyclonal/PA5-79129).
Anti-TH: this antibody has been used in Western blot and IHC to detect TH in the mouse brain (Ki et al. eLife 10:e70920 (2021)).
Anti-mCherry: this antibody has been widely used for detecting recombinant mCherry (https://www.abcam.com/mcherry-antibody-ab167453.html)
Anti-S100b: many studies have used this antibody to probe S100b in mice (see, Pattwell et al. Nat Commun 7: 11475 (2016)).
Anti-GluA1: this antibody detects GluA1 in rat and mice. This has been used in our previous publication and others (Li et al. Proc Natl Acad Sci U S A: 13(11):E1575-84(2016)).
Anti-GluA2: it has been validated in mouse brain, as shown on the manufacturer's website (https://www.thermofisher.com/antibody/product/GluR2-Antibody-Polyclonal/PA5-19496)
Anti-GluA2: it has been validated in mouse brain, as shown on the manufacturer's website (https://www.thermofisher.com/antibody/product/GluR2-Antibody-clone-7G6-Monoclonal/MA5-17084).
Anti-GluA4: this antibody has been validated in mice using IHC and Western blotting, as shown on the manufacturer's website (https://www.sigmaaldrich.com/US/en/product/mm/ab1508).
Anti-p831 GluA1: this antibody detects p831 GluA1 in mice. This has been used in Western blot in our previous publication (Li et al. Proc Natl Acad Sci U S A: 13(11):E1575-84(2016)).
Anti-p845 GluA4: this antibody detects p845 GluA1 in mice. This has been used in Western blot in our previous publication (Li et al. Proc Natl Acad Sci U S A: 13(11):E1575-84 (2016)).
Anti-c-fos: this antibody has been validated in mice in previous publications (Pan et al. Cell Rep 36:109398 (2021)).
Anti-PV: this antibody has been validated in mice by IHC, as shown on the manufacturer's website (https://sysy.com/product/195004#gallery-3).

Anti-mTOR: applications of this antibody include IHC and Western blotting. It detects mTOR in the mouse brain and has been previously validated (Gnanapradeepan et al. Elife ;9:e55994 (2020)).
Anti-p2448 mTOR: this antibody can be used in IHC and Western blot to detect p2448 mTOR in mice and has been successfully used (Yuskaitis et al. Cell Rep 40:111278 (2022)).
Anti-b-actin: this antibody has been used to detect b-actin, as shown on the vendor's website (https://www.thermofisher.com/antibody/product/beta-Actin-Loading-Control-Antibody-clone-BA3R-Monoclonal/MA5-15739).

Animals and other research organisms

Policy information about studies involving animals; ARRIVE guidelines recommended for reporting animal research, and Sex and Gender in Research

Laboratory animals	Both male and female animals at age of 2-3 months were used in this study. Mouse strains are as follows:<br>B6.Cg-Tg(Drd1a-tdTomato)6Calak/J (Jackson Laboratory);<br>Tg(Drd2-EGFP)S118Gsat/Mmnc (MMRRC);<br>B6.SJL-Slc6a3tm1.1(cre)Bkmn/J (Jackson Laboratory);<br>B6.129-Tg(Pcp2-cre)2Mpin/J (Jackson Laboratory);<br>Tg(Slc1a3-cre/ERT)1Nat/J (Jackson Laboratory);<br>Drd1tm2.1Stl/J (Jackson Laboratory).<br>Drd1 cKO (cross Tg(Slc1a3-cre/ERT)1Nat/J mice with Drd1tm2.1Stl/J mice; tamoxifen was administered to their offspring)
Wild animals	This study did not involve wild animals.
Reporting on sex	We used both male and female mice in our study. Our initial observation did not find the difference between two sexes. We pooled data from both sexes for analyses.
Field-collected samples	This study did not involve samples collected from the field.
Ethics oversight	All experimental protocols involving the use of animals were reviewed and approved annually by the Institutional Animals Care and Use Committee of the University of Alabama at Birmingham (IACUC-22247).

Note that full information on the approval of the study protocol must also be provided in the manuscript.
